# Supplementary material for: A novel one-step quick assay for detection of SARS-COV2 antibodies across mammalian species
Source: PeerJ. 2021 Apr 27;9:e11381. doi: 10.7717/peerj.11381 (PMC8086566; doi:10.7717/peerj.11381)
Supplement: Supplemental Information 1 — 50 pre-pandemic human serum samples were purchased from San Diego Blood Bank in July, 2019. Sample IDs were renamed to protect identifiable information. 40 SARS-CoV2 patient serum samples were purchased from RayBiotech. Sample IDs were renamed and age ranges were provided to protect patient identifiable information. All of the patients were diagnosed by PCR except one with antigen diagnosis. There are 5 PCR negative patients, but they appear to have antibodies against SARS-CoV2 RBD. The only negative sample by the one-step test is B14 that was PCR negative and has low levels of antibodies against SARS-CoV2 RBD. [file peerj-09-11381-s001.docx]

**Supplemental Material**

50 pre-pandemic human serum samples were purchased from San Diego Blood Bank in July, 2019. Sample IDs were renamed to protect identifiable information.

40 SARS-CoV2 patient serum samples were purchased from RayBiotech. Sample IDs were renamed and age ranges were provided to protect patient identifiable information. All of the patients were diagnosed by PCR except one with antigen diagnosis. There are 5 PCR negative patients, but they appear to have antibodies against SARS-CoV2 RBD. The only negative sample by the one-step test is B14 that was also PCR negative.
