# Supplementary figures and images for: A novel one-step quick assay for detection of SARS-COV2 antibodies across mammalian species
Source: PeerJ. 2021 Apr 27;9:e11381. doi: 10.7717/peerj.11381 (PMC8086566; doi:10.7717/peerj.11381)

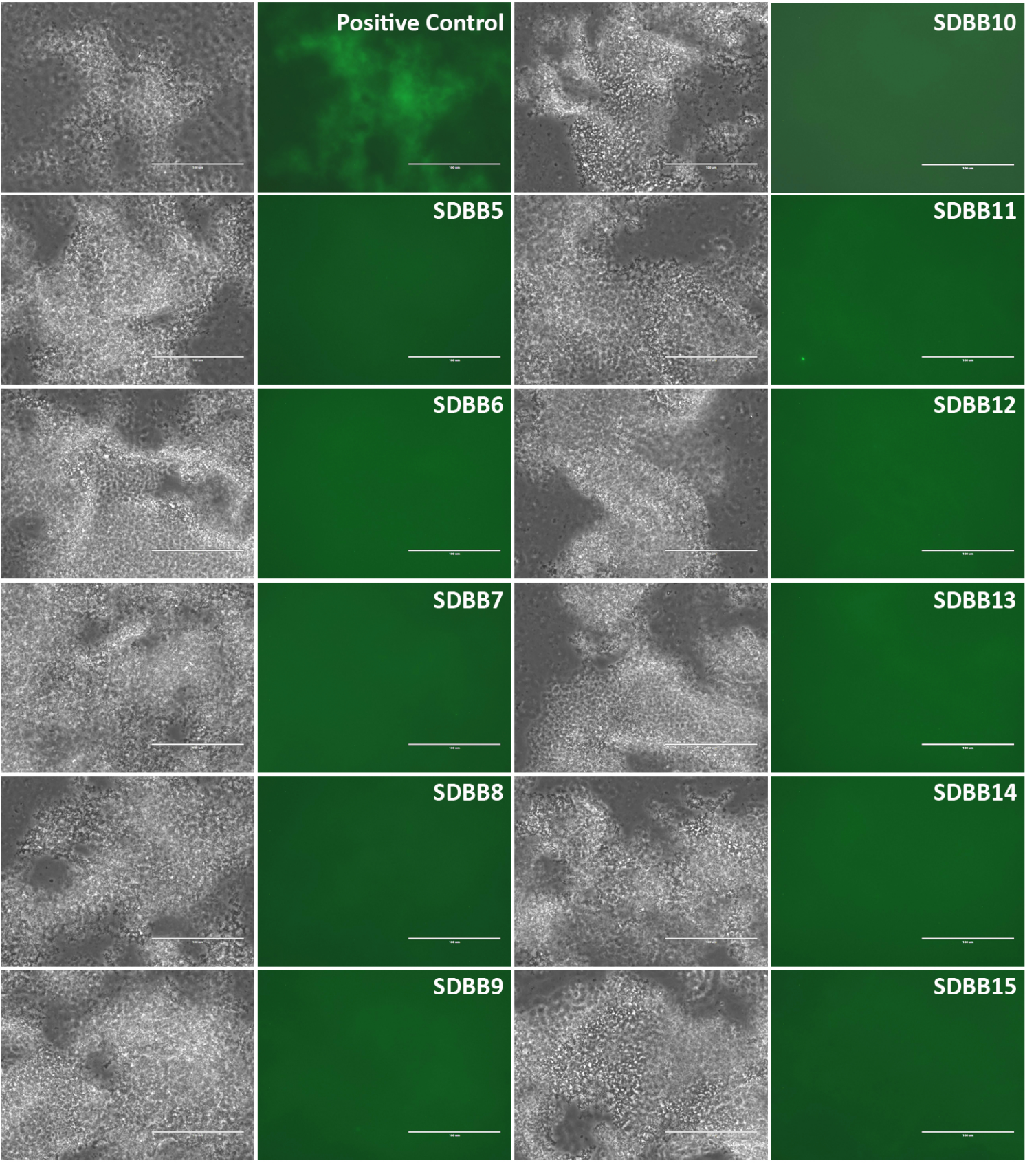

**Fig. S1**

Supplement: Supplemental Information 4 — The positive control is rabbit anti-scN antibodies diluted in human serum. [file peerj-09-11381-s004.pdf]

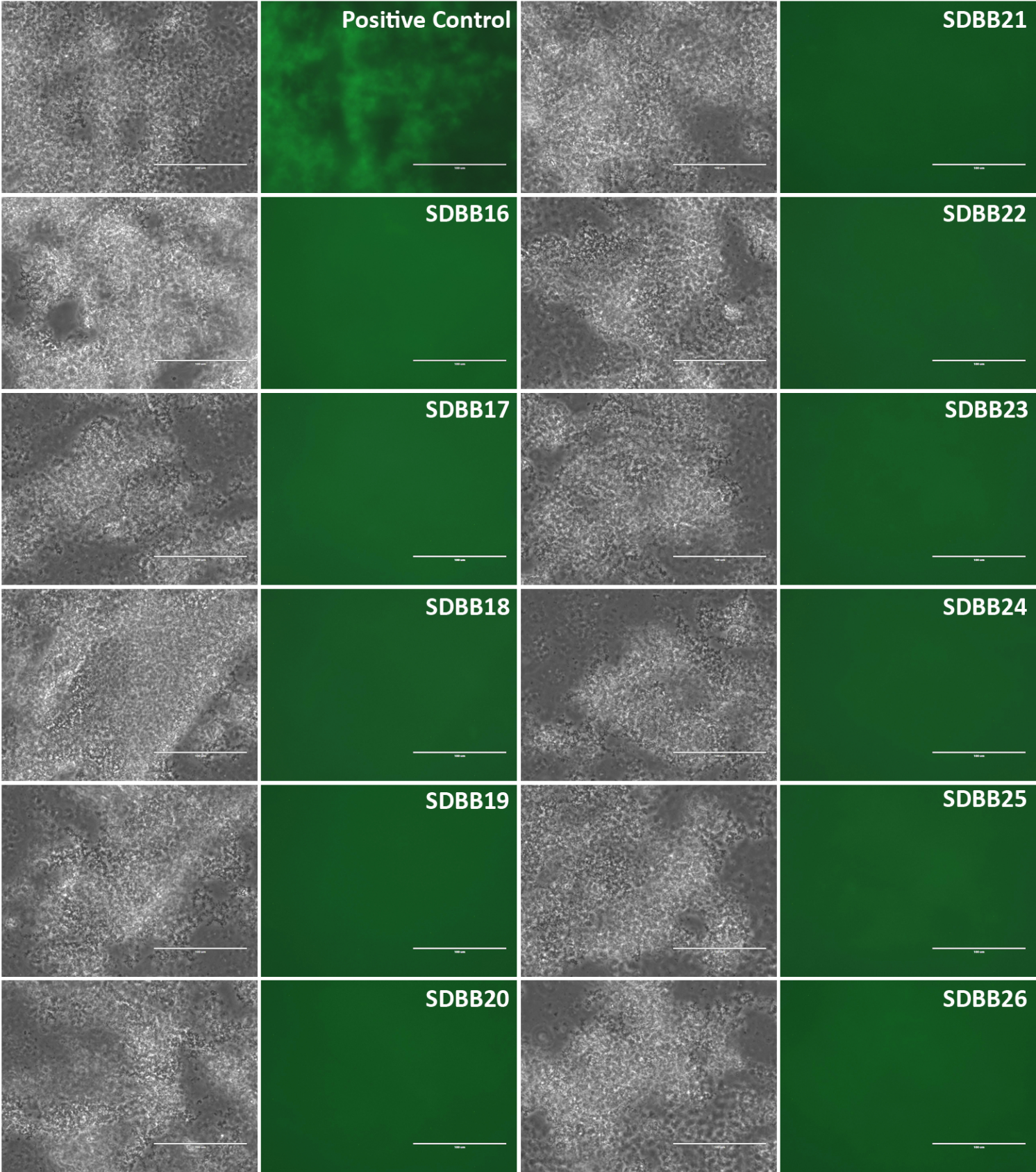

**Fig. S2**

Supplement: Supplemental Information 5 — The positive control is rabbit anti-scN antibodies diluted in human serum. [file peerj-09-11381-s005.pdf]

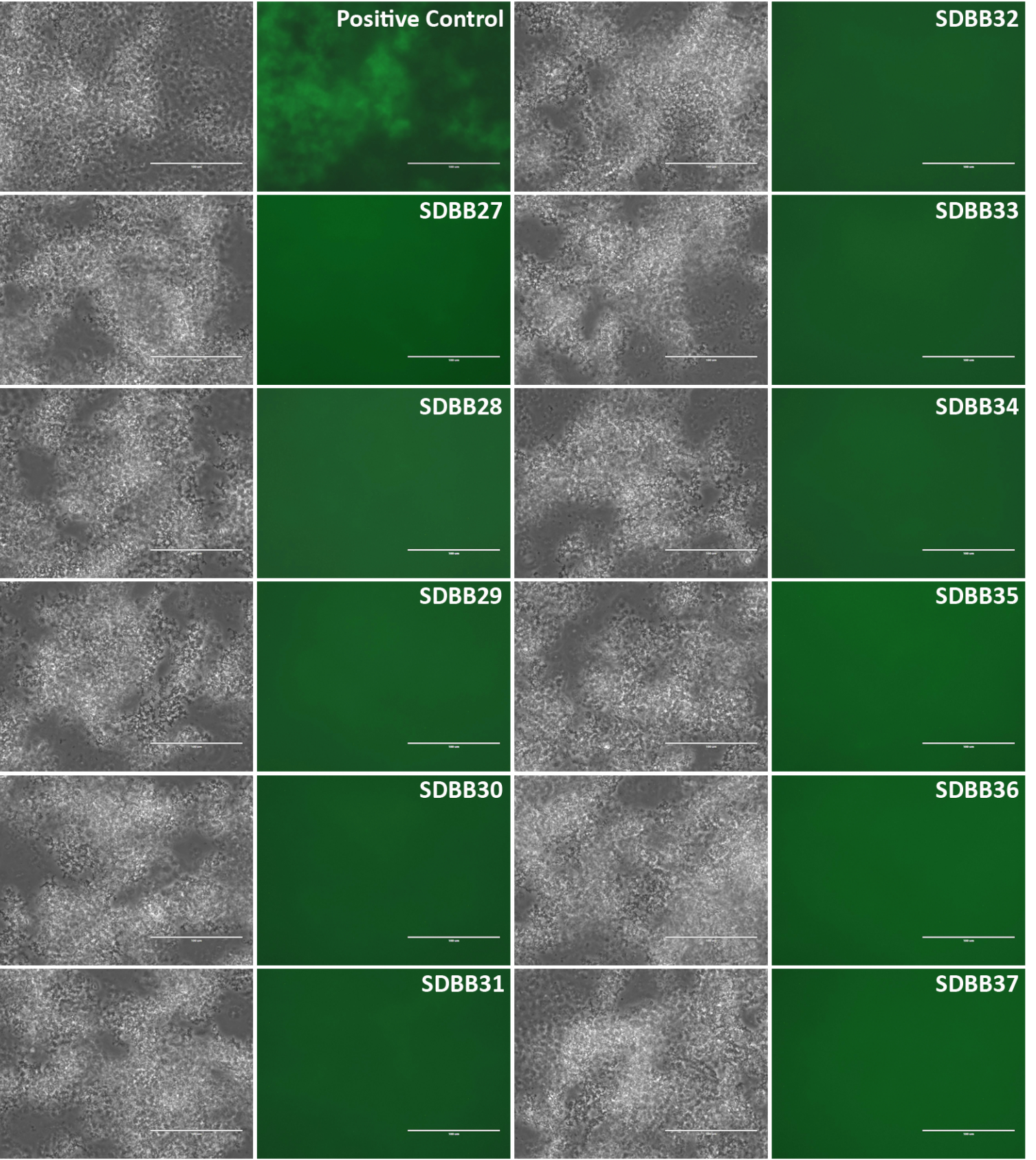

**Fig. S3**

Supplement: Supplemental Information 6 — The positive control is rabbit anti-scN antibodies diluted in human serum. [file peerj-09-11381-s006.pdf]

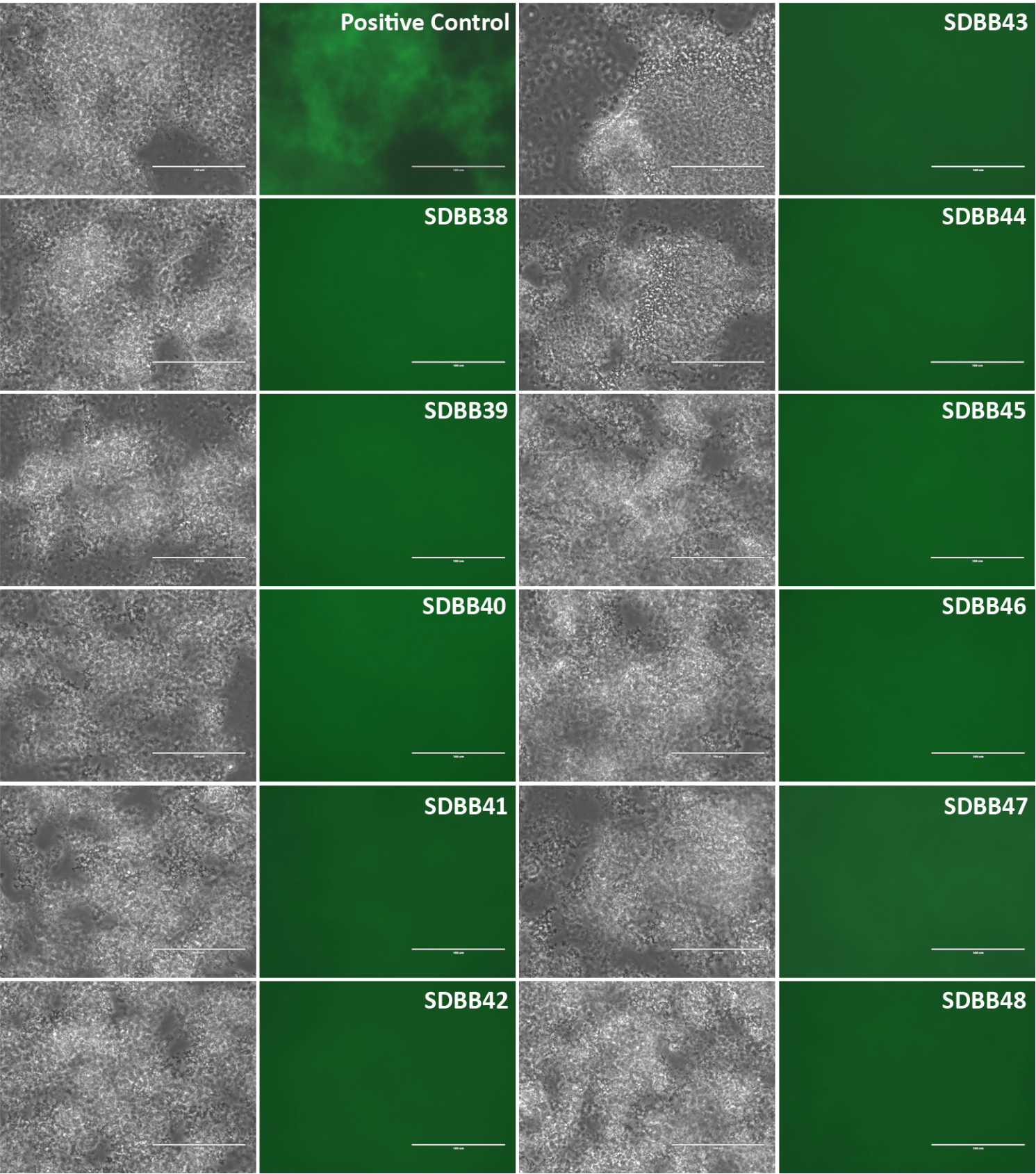

**Fig. S4**

Supplement: Supplemental Information 7 — The positive control is rabbit anti-scN antibodies diluted in human serum. [file peerj-09-11381-s007.pdf]

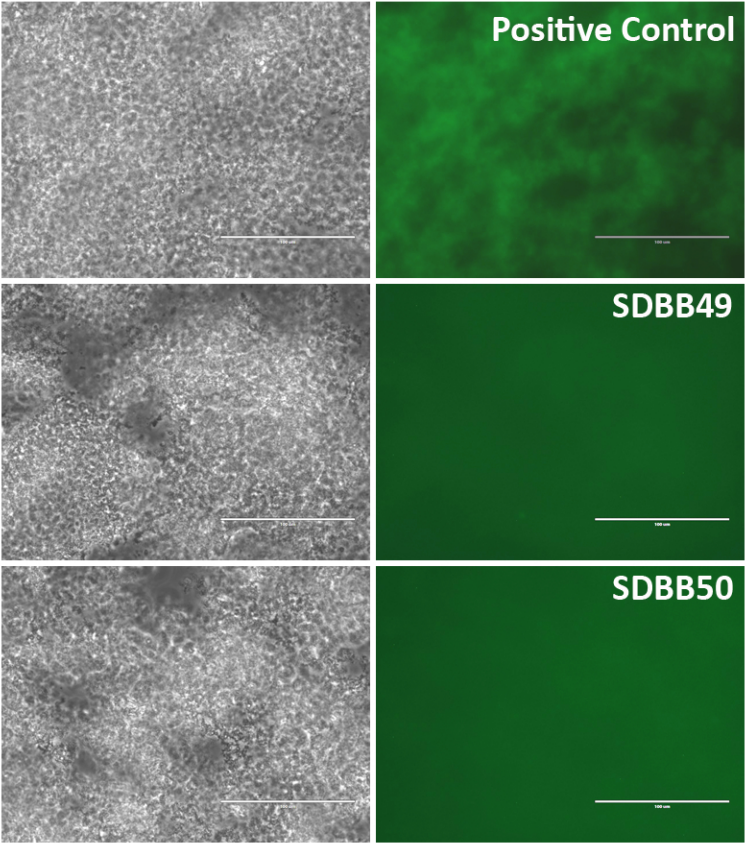

**Fig. S5**

Supplement: Supplemental Information 8 — The positive control is rabbit anti-scN antibodies diluted in human serum. [file peerj-09-11381-s008.pdf]

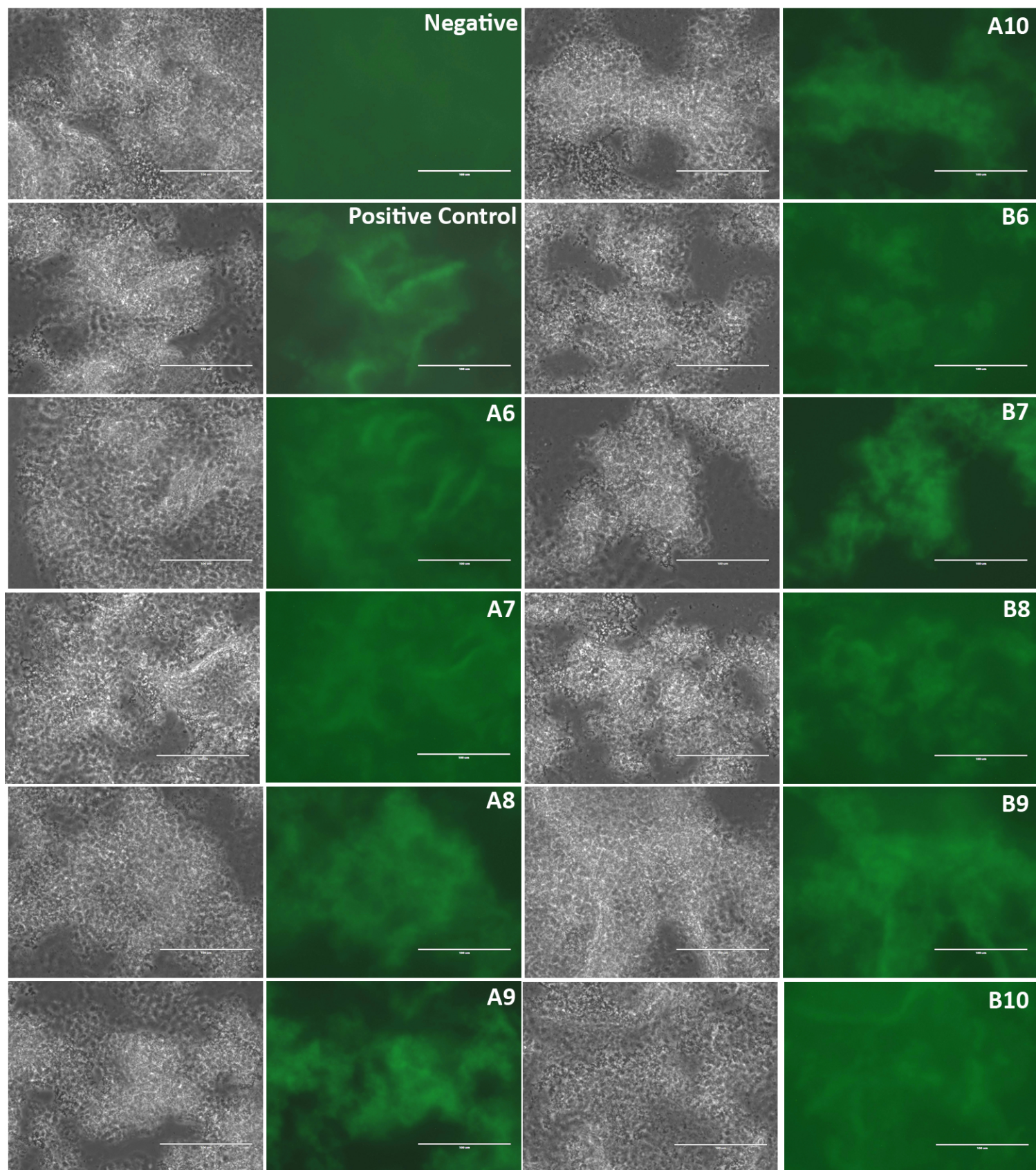

**Fig. S6**

Supplement: Supplemental Information 9 — Raw data from sera of patients A6-10, B6-10 from RayBiotech. The positive control is rabbit anti-scN antibodies diluted in human serum. The negative control is pre-pandemic serum from San Diego Blood Bank. [file peerj-09-11381-s009.pdf]

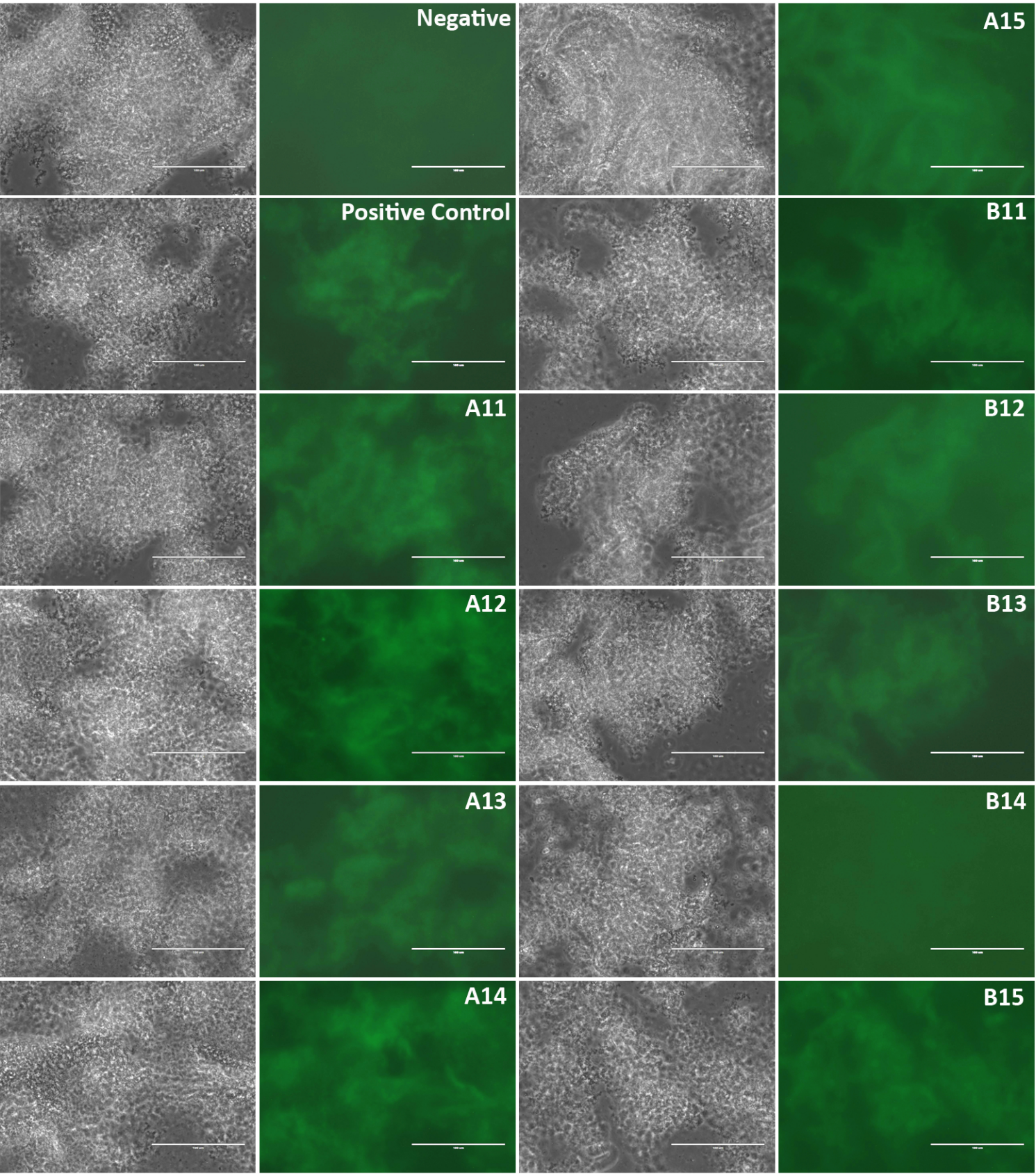

**Fig. S7**

Supplement: Supplemental Information 10 — The positive control is rabbit anti-scN antibodies diluted in human serum. The negative control is pre-pandemic serum from San Diego Blood Bank. [file peerj-09-11381-s010.pdf]

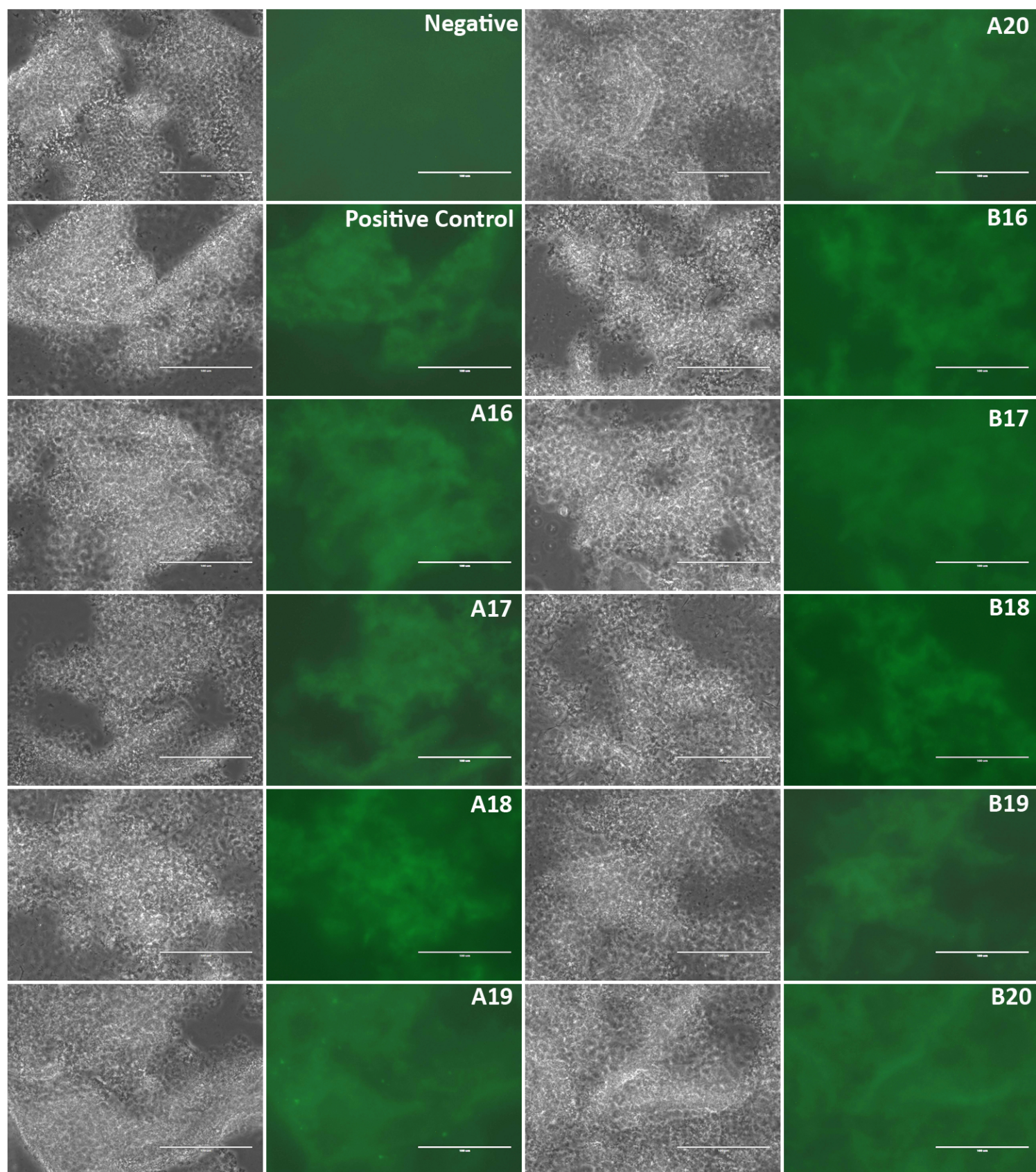

**Fig. S8**

Supplement: Supplemental Information 11 — The positive control is rabbit anti-scN antibodies diluted in human serum. The negative control is pre-pandemic serum from San Diego Blood Bank. [file peerj-09-11381-s011.pdf]
